# Hypnosis as a treatment of chronic widespread pain in general practice: A randomized controlled pilot trial

**DOI:** 10.1186/1471-2474-9-124

**Published:** 2008-09-18

**Authors:** Jan Robert Grøndahl, Elin Olaug Rosvold

**Affiliations:** 1 Institute of General Practice and Community Medicine, Faculty of Medicine, University of Oslo, Norway; 2 Tranby Legesenter, 3408 Tranby, Norway

## Abstract

**Background:**

Hypnosis treatment in general practice is a rather new concept. This pilot study was performed to evaluate the effect of a standardized hypnosis treatment used in general practice for patients with chronic widespread pain (CWP).

**Methods:**

The study was designed as a randomized control group-controlled study. Sixteen patients were randomized into a treatment group or a control group, each constituting eight patients. Seven patients in the treatment group completed the schedule. After the control period, five of the patients in the control group also received treatment, making a total of 12 patients having completed the treatment sessions. The intervention group went through a standardized hypnosis treatment with ten consecutive therapeutic sessions once a week, each lasting for about 30 minutes, focusing on ego-strengthening, relaxation, releasing muscular tension and increasing self-efficacy. A questionnaire was developed in order to calibrate the symptoms before and after the 10 weeks period, and the results were interpolated into a scale from 0 to 100, increasing numbers representing increasing suffering. Data were analyzed by means of T-tests.

**Results:**

The treatment group improved from their symptoms, (change from 62.5 to 55.4), while the control group deteriorated, (change from 37.2 to 45.1), (p = 0,045). The 12 patients who completed the treatment showed a mean improvement from 51.5 to 41.6. (p = 0,046). One year later the corresponding result was 41.3, indicating a persisting improvement.

**Conclusion:**

The study indicates that hypnosis treatment may have a positive effect on pain and quality of life for patients with chronic muscular pain. Considering the limited number of patients, more studies should be conducted to confirm the results.

**Trial Registration:**

The study was registered in ClinicalTrials.gov and released 27.08.07 Reg nr NCT00521807 Approval Number: 05032001.

## Background

Chronic widespread pain (CWP) is defined by the classification criteria of The American College of Rheumatology from 1990 as pain during at least four of the days of the week lasting for at least three months, localized both over and below the waist, and on both the left and right side of the body, as well as in the back and neck [1]. If there are at least 11 out of 18 defined positive trigger points, the condition will be defined as fibromyalgia (FM).

Pain in the muscular-skeleton system is common both in Norway and in the northern hemisphere. In one Norwegian study, 22% out of 2664 randomly selected women reported chronic widespread muscular pain, while 25% reported chronic localized muscular pain [2]. Another study of Norwegian women aged 20–50 years found a prevalence of FM and CWP on 10.5% and 25%, respectively [3].

It appears that muscular pain is representing a continuous spectrum from just a little pain to chronic debilitating pain. One study concludes: "There is little evidence that fibromyalgia or chronic, widespread pain comprises a distinct entity. It is more likely that these criteria select persons at one end of a continuum of pain, from humdrum nuisance to chronic, disabling disease" [4]. Both physical and psychological factors are believed to be involved in the development of chronic muscular pain, and it is supposed that it exist individual differences in sensitivity and tolerance for various stimuli [5,6].

The treatment offered at present to patients with CWP ranges from physiotherapy, analgetic drugs, advices about varied physical activity both at work and in leisure time, psychomotoric physiotherapy, consulting therapy and antidepressant medications. Over the years the patients may have slight variations in their degree of suffering, although the symptoms usually stay fairly unchanged over time [7]. The condition seems to have both biological and psychological causes. Hence a treatment like hypnosis, aiming both at improving mind-body control and psychological mastering, seems theoretically rational.

One experimental study including forty-five patients with fibromyalgia finds that hypnosis followed by analgesia suggestions has a greater effect on the intensity of pain and on the sensory dimension of pain than hypnosis followed by relaxation suggestions; and that the effect of hypnosis followed by relaxation suggestions is not greater than relaxation [8]. This indicates that hypnotic treatment might influence physiologic responses through mental processes.

In another study the researchers measured regional cerebral blood flow with positron emission tomography in patients with fibromyalgia, during hypnotically-induced analgesia and resting wakefulness [9]. The patients experienced less pain during hypnosis than at rest, and the blood-flow pattern of the brain was notably changed, supporting notions of a multifactorial nature of hypnotic analgesia, with an interplay between cortical and subcortical brain dynamics.

Therapeutic use of hypnosis is not common in the northern hemisphere, and has only to a small extent been tested in relation to chronic muscular pain. A review of 13 controlled prospective trials of hypnosis for the treatment of chronic pain indicate that hypnosis interventions consistently produce significant decreases in pain associated with a variety of chronic-pain problems [10]. However, the authors comment that there is a lack of standardization of the hypnotic interventions examined in clinical trials, and that the number of patients enrolled in the studies has tended to be low and lacking long-term follow-up.

One Norwegian study that used guided imagery found reduction of fibromyalgic pain during the study period [11]. In a German trial it was not possible to measure an effect of hypnosis on chronic pain [12]. In a Dutch controlled study hypnotherapy was found to reduce pain experience in patients with refractory fibromyalgia, but this was not reflected in an improvement of the total myalgic score measured by a dolorimeter [13]. The authors conclude that further studies should be conducted with patients having a shorter history of disease.

The present study was conducted to develop and document the effect of a standardized hypnosis treatment used in general practice for patients with CWP.

## Methods

**Figure 1:**
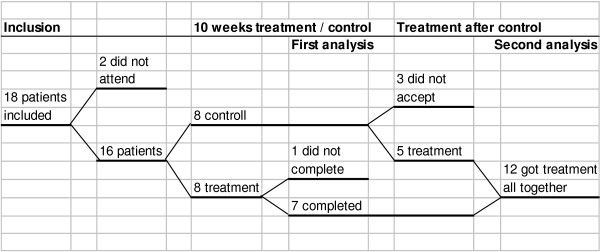
Float diagram of participants in the study.

The study is a randomized, controlled study, and was conducted during 2001 – 2003. Eighteen patients having had CWP for at least three months and at most five years were recruited from the main authors' own general practice, or from colleagues working in the same area. Patients having primarily other organic diseases or serious psychiatric disorders were excluded. Two of the patients did not attend the first session, and the remaining 16 patients, 12 women and four men, aged 23–54 years, were randomized into an intervention group and a control group. In the 10 week intervention period both groups were offered similar treatments according to normal routines in general practice, with the addition of hypnosis treatment in the intervention group. The treatments included different combinations of medications such as analgesics and antidepressants, and physiotherapy or chiropractic therapy. One patient in the intervention group did not continue through the whole treatment schedule, but is included in the material in accordance with the statistical principle of intention to treat [14]. Hence there were seven patients completing the treatment during the first phase (Figure 1). After the 10 week period, all the eight patients in the control group were offered the hypnosis treatment, and as five of them accepted, a total of 12 patients completed the treatment.

**Figure 2:**
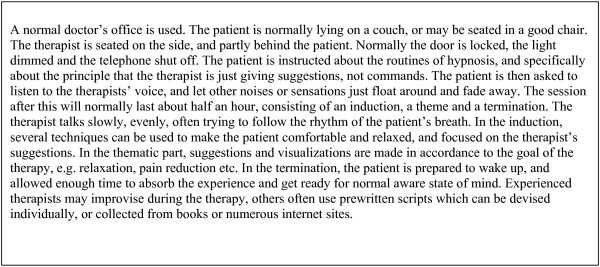
Hypnosis in general practice – how is it done?

The intervention group went through a standardized hypnosis treatment once a week for ten weeks. The treatment was performed by the main author, being a general practitioner with special interest and training in hypnosis. Hypnosis is a treatment where the patient is inducted into a slightly altered state of consciousness, still being alert and awake, but more distanced from the outer world, and more focused on his or her inner thoughts and emotions [15]. Each therapeutic session lasted for about 30 minutes, focusing on ego-strengthening, relaxation, releasing muscular tension and increasing the self-efficacy. Visualization techniques were used to improve self-evaluation and to create a more positive body experience. The content of every therapeutic session was connected to the previous one, in order to make the patient more comfortable and able to relax during the treatment. The treatment was based on a manual which was developed for the study, prescribing every treatment session in detail, and followed rigorously and in the same order for every patient. Every hypnosis session was recorded on audio tape, which the patient kept for use at home before the next session. More details about hypnosis in general practice is given in Figure 2

The treatment performed in this study was, like most hypnosis performed by professionals, based on permissive suggestions in conformation with the patients consent. Of the present two mainstreams of theoretical basis for hypnosis, the more individualistic ideas of Milton H. Ericson were introduced to all the patients suggesting that their experience during hypnosis is unique and valid without necessity for objective evaluation or scaling [15]. On the other hand, the actual treatment was more in the line of Ernest G. Hilgard's theories in the sense that a standard treatment was given to all the participants, without individual adjustments [15]. This is in accordance with our aim to make a standardized tool but still respecting the individualistic nature of the experience of hypnosis.

A questionnaire was developed as to measure the patients' symptoms, consisting of 25 questions divided into five main sections (Additional file 1). The first section concerned pain at activity and rest, fatigue and concentration problems, the second was dealing with activities of daily life, like dressing, carrying groceries, walking and running. The third section was one question concerning subjective evaluation of quality of life in total. The fourth was an estimate of how much the pain interfered with work, hobbies and social life, and the fifth was an estimate of feelings of inadequacy, anxiety, loneliness and pessimism.

The questionnaire contained elements from WHOQOL-BREF – a questionnaire concerning quality of life developed by WHO [16], from SHC – Subjective Health Complaint inventory developed by Ursin et al [17], and from Hopkins Symptom Checklist (SCL-5) [18].

The results were interpolated into a scale from 0 to 100, increasing numbers representing increasing suffering (Additional file 2).

All participants answered the questionnaire at the time of inclusion, and again after 10 weeks. Those patients receiving treatment after the control period also answered the same questionnaire once more after the treatment. In addition, all the 12 patients who received treatment filled in the same questionnaire one year later, including an additional question on use of the audio-tapes from the hypnosis sessions.

Data analysis (T-tests) was performed with SPSS version 12. The p-value was set to p ≤ 0.05. The study was approved by the Norwegian Southern Regional Committee for Medical Research Ethics. All patients were given written information about the study at the time of inclusion, and their oral consent to participate was noted in their journals.

The study was registered in ClinicalTrials.gov and released 27.08.07 Reg nr NCT00521807 Approval Number: 05032001. It was registered after it was completed, since registration of clinical trials was not so prevalent at the time it was carried out.

## Results

The seven patients in the treatment group showed an average improvement in scores of -7.1 from 62.5 to 55.4, whilst the eight patients in the control group had a deterioration of 7.9 from 37.2 to 45.1. A T-test showed the difference between the groups to be statistically significant (p = 0.045).

**Figure 3:**
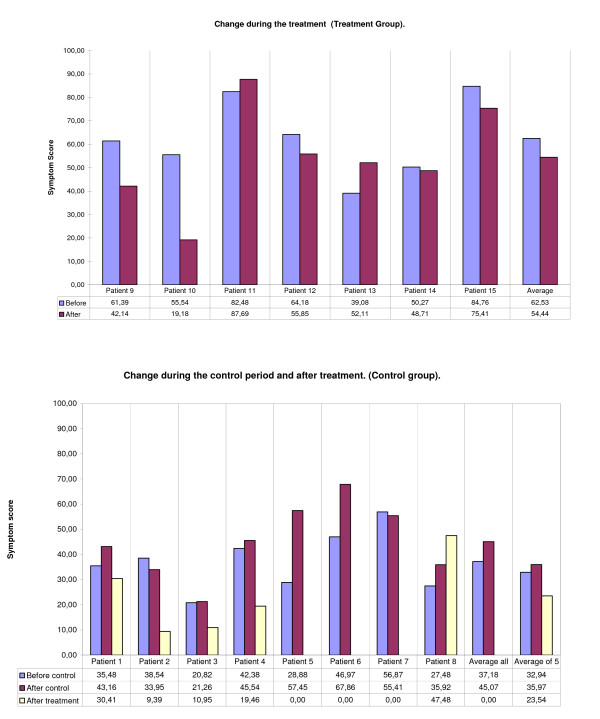
**Symptom score before and after the treatment period in the treatment group and before and after the control period in the control group, (Blue and purple columns)**. The results for the five patients in the control group who later received treatment is included (white column).

The five patients who received hypnosis treatment after first having been part of the control group had an average improvement of -12.43 from 35.97 to 23.54. The scores for the individual patients are shown in Figure 3.

The total of 12 patients who completed the treatment showed a mean improvement of -9.9 from 51.5 to 41.6. It was done an estimate from a zero-hypothesis, since there was no longer any control group to compare with. A one-sample T-test on this material shows the improvement to be statistically significant (p = 0.046).

All the 12 patients who received treatment answered the questionnaire one year later. During treatment they showed a mean improvement from 51.5 to 41.6, and after a year the corresponding result was 41.3, indicating that the improvement maintained at least for one year.

All the 12 patients reported that they used the audio tapes they had received, or some other kind of auto-hypnosis, at least once weekly, and three reported almost daily use. All of the patients also reported that they most probably would have accepted more hypnosis treatment, if available.

## Discussion

The study indicates that hypnosis treatment in general practice for patients with CWP may have positive effects, and that the effect persists over time.

Due to the small number of patients, large changes are required to make statistically significant results. Nevertheless, our analyses indicate statistically significant changes during and after treatment. It seems that some of the patients benefited strongly from the treatment, and these are the major contributors to the positive results of the study. This emphasizes the vulnerability of such a small study, but it also raises the question as to whether the variations is caused by different aetiology of the disease, or by unequal hypnotic susceptibility.

In spite of being adequately randomized, the two groups initially were very different according to their level of suffering: the treatment group starting at 62.5 and the control group at 37.2. This raises a question as to whether the part of the study based on the comparison between groups is reliable. The fact that the treatment group was worse off at the start, also raises the question as to whether the results are due to regression to the mean, indicating that there is a tendency of the extremes to normalize [19]. This, however, does not seem to be the case for the five who received treatment after first having been a part of the control-group. Even though starting at a comparable low level of suffering, they improved considerably during treatment.

This kind of study may be biased by the patients' wishes to give good evaluations in order to please their therapist, which will tend to influence the results in a positive direction. In the treatment period we tried to avoid the personal aspect by emphasizing to every paritisipant that the results were anonymus also to the terapist, as the questionnaires were collected by the other author.

The questionnaire was developed specifically for this study. It encompasses the parameters that the study was designed to investigate, and is partly based on other, validated questionnaires, but it has not itself been scientifically validated. This dictates the need for caution in interpretation of the results. However, this pilot study indicates that further studies on hypnotic treatment for muscular-skeletal diseases and pain might be rewarding. Only two controlled studies were found on a PubMed search using the words hypnosis, general practice and trial or study [20,21]. Further studies clearly should include more patients, and validated questionnaires should be used.

Since the hypnosis treatment was not specifically designed to relieve the symptoms of CWP, it would be interesting to study the effect of a similar treatment for other kinds of suffering, i.e. chronic rheumatic diseases, chronic pain conditions, and psychical diseases like anxiety and depression.

The effect of the treatment will depend to some extent on the experience of the therapist, and also on the relevance of the suggestions and visualizations that are given. In this respect the treatment can still be improved further, in order to maximize the effect.

Recording of each therapeutic session makes way for rationalizing the treatment, for example producing all the sessions as CD-recordings combined with less time and effort-demanding counselling by the therapist. Most patients reported, however, that the live treatment done by the therapist was the most effective. Use of recordings versus live sessions should therefore be investigated in further detail.

Provided with recordings of the treatment sessions, it is also probable that the treatment could be copied by other general practitioners, who would then be able to perform the treatment after some specific directed training.

## Conclusion

The study indicates that hypnosis treatment may have a positive effect on pain and quality of life for patients with chronic muscular pain The effect seems to persist for at least one year. Considering the limited number of patients, more studies should be conducted to confirm the results.

## Competing interests

The authors declare that they have no competing interests.

## Authors' contributions

JRG designed the study and the questionnaire, performed the hypnoses treatment and the statistical analyses and drafted the manuscript. EOR participated in the data collection and in the statistical analyses and drafting of the manuscript. Both authors read and approved the final manuscript.

## Pre-publication history

The pre-publication history for this paper can be accessed here:



## Supplementary Material

Additional file 1 **Table 1. Summary of questionnaire.** Click here for file

Additional file 2 **Table 2. Calculation of scores, explanation and example.** Click here for file
