# Supplementary material for: Hypnosis as a treatment of chronic widespread pain in general practice: A randomized controlled pilot trial
Source: BMC Musculoskelet Disord. 2008 Sep 18;9:124. doi: 10.1186/1471-2474-9-124 (PMC2553788; doi:10.1186/1471-2474-9-124)
Supplement: Additional file 1 — Table 1. Summary of questionnaire. [file 1471-2474-9-124-S1.doc]

## Table 1: Summary of questionnaire

| **1) Degree of symptoms last week** | | | | |  | | |  |  |  |  |  |  |  |  |  |  |  |
| --- | --- | --- | --- | --- | --- | --- | --- | --- | --- | --- | --- | --- | --- | --- | --- | --- | --- | --- |
|  | **Pain on activity** | |  | | |  | |  |  |  |  |  |  |  |  |  |  |  |
|  | **Pain at rest** | |  | | |  | |  |  |  |  |  |  |  |  |  |  |  |
|  | **Tiredness** | |  | | |  | |  |  |  |  |  |  |  |  |  |  |  |
|  | **Concentration problems** | | | |  | | |  |  |  |  |  |  |  |  |  |  |  |
|  |  |  | |  | | | 1 | 2 | 3 | 4 | 5 | 6 | 7 | 8 | 9 |  |  |  |
|  |  |  | |  | | | None | |  |  |  |  |  |  | Strongest possible | | | |
|  |  |  | |  | | |  |  |  |  |  |  |  |  |  |  |  |  |
| **2) Functions, able to:** | | |  | | |  | |  |  |  |  |  |  |  |  |  |  |  |
| **Put on shoes, stockings** | | |  | | |  | |  |  |  |  |  |  |  |  |  |  |  |
| **Carry 5kg in a shopping bag 30m** | | |  | | |  | |  |  |  |  |  |  |  |  |  |  |  |
| **Collect something from a high cupboard** | | | | |  | | |  |  |  |  |  |  |  |  |  |  |  |
| **Clean the windows** | |  | |  | | |  |  |  |  |  |  |  |  |  |  |  |  |
| **Run 100m without stopping due to pain** | | | | |  | | |  |  |  |  |  |  |  |  |  |  |  |
| **Walk 400m without stopping due to pain** | | | | |  | | |  |  |  |  |  |  |  |  |  |  |  |
|  |  |  | |  | | | 1 | 2 | 3 | 4 | 5 | 6 | 7 |  |  |  |  |  |
|  |  |  | |  | | | No problem | | | |  |  | Not at all able to | | | | |  |
|  |  |  | |  | | |  |  |  |  |  |  |  |  |  |  |  |  |
| **3) Subjective quality of life right now** | | | | |  | | |  |  |  |  |  |  |  |  |  |  |  |
|  |  |  | |  | | |  |  |  |  |  |  |  |  |  |  |  |  |
|  |  |  | |  | | | 1 | 2 | 3 | 4 | 5 | 6 | 7 | 8 | 9 | 10 |  |  |
|  |  |  | |  | | | Worst possible | | | | |  |  |  |  | Best possible | |  |
|  |  |  | |  | | |  |  |  |  |  |  |  |  |  |  |  |  |
| **4) I feel physically weakened or restrained** | | | | | | | | |  |  |  |  |  |  |  |  |  |  |
|  | By disease | |  | | |  | |  |  |  |  |  |  |  |  |  |  |  |
|  | By pain |  | |  | | |  |  |  |  |  |  |  |  |  |  |  |  |
|  | By tension in my body | | | |  | | |  |  |  |  |  |  |  |  |  |  |  |
|  | In my daily activities | | | |  | | |  |  |  |  |  |  |  |  |  |  |  |
|  | In my work | |  | | |  | |  |  |  |  |  |  |  |  |  |  |  |
|  | In my leisure time | |  | | |  | |  |  |  |  |  |  |  |  |  |  |  |
|  | In my social life | |  | | |  | |  |  |  |  |  |  |  |  |  |  |  |
|  |  |  | |  | | | 1 | 2 | 3 | 4 | 5 | 6 | 7 |  |  |  |  |  |
|  |  |  | |  | | | Not at all | | |  |  |  | Very much | | | |  |  |
|  |  |  | |  | | |  |  |  |  |  |  |  |  |  |  |  |  |
| **5) I feel (psychically)** | | |  | | |  | |  |  |  |  |  |  |  |  |  |  |  |
|  | Insufficient | |  | | |  | |  |  |  |  |  |  |  |  |  |  |  |
|  | Scared, anxious | |  | | |  | |  |  |  |  |  |  |  |  |  |  |  |
|  | Hopelessness and depression | | | |  | | |  |  |  |  |  |  |  |  |  |  |  |
|  | Bad consciousness | | | |  | | |  |  |  |  |  |  |  |  |  |  |  |
|  | Lonely |  | |  | | |  |  |  |  |  |  |  |  |  |  |  |  |
|  | Scared of the future | | | |  | | |  |  |  |  |  |  |  |  |  |  |  |
|  | Indifference | |  | | |  | |  |  |  |  |  |  |  |  |  |  |  |
|  |  |  | |  | | | 1 | 2 | 3 | 4 | 5 | 6 | 7 |  |  |  |  |  |
|  |  |  | |  | | | Not at all | | |  |  |  | Very much | | | |  |  |
|  |  |  | |  | | |  |  |  |  |  |  |  |  |  |  |  |  |
