# Supplementary material for: Hypnosis as a treatment of chronic widespread pain in general practice: A randomized controlled pilot trial
Source: BMC Musculoskelet Disord. 2008 Sep 18;9:124. doi: 10.1186/1471-2474-9-124 (PMC2553788; doi:10.1186/1471-2474-9-124)
Supplement: Additional file 2 — Table 2. Calculation of scores, explanation and example. [file 1471-2474-9-124-S2.doc]

## Table 2: Calculation of scores, explanation and example

| **1) Degree of symptoms last week** | | | | |  |  |  |  |  |  |  |  |  |  |
| --- | --- | --- | --- | --- | --- | --- | --- | --- | --- | --- | --- | --- | --- | --- |
|  | 4 questions, answer from best 1 to worst 9. | | | |  | **Average** | | |  | 4,25 |  |  |  |  |
|  |  |  |  |  |  |  |  |  |  |  |  |  |  |  |
| **2) Functions, able to:** | | |  |  |  |  |  |  |  |  |  |  |  |  |
|  | 6 questions, answer from best 1 to worst 7. | | | |  | **Average** | | |  | 2,17 |  |  |  |  |
|  |  |  |  |  |  |  |  |  |  |  |  |  |  |  |
| **3) Subjective quality of life right now.** | | | | |  |  |  |  |  |  |  |  |  |  |
|  | 1 question, answer from worst 1 to best 10 | | | |  |  |  |  |  |  |  |  |  |  |
|  | Reversed: 11- Given answer | |  |  |  | **Reversed** | | | | 5,00 |  |  |  |  |
|  |  |  |  |  |  |  |  |  |  |  |  |  |  |  |
| **4) I feel physically weakened or restrained.** | | | | |  |  |  |  |  |  |  |  |  |  |
|  | 7 questions, answer from best 1 to worst 7. | | | |  | **Average** | | |  | 3,57 |  |  |  |  |
|  |  |  |  |  |  |  |  |  |  |  |  |  |  |  |
| **5) I feel (psychically)** | | |  |  |  |  |  |  |  |  |  |  |  |  |
|  | 7 questions, answer from best 1 to worst 7. | | | |  | **Average** | | |  | 2,43 |  |  |  |  |
|  |  |  |  |  |  |  |  |  |  |  |  |  |  |  |
|  |  |  |  |  |  |  |  |  |  |  |  |  |  |  |
|  | **Average of the 5 main sections:** | | |  | **Total average** | | | | | 3,48 |  |  |  |  |
|  | (The total average will be in the range of between minimum | | | | | |  |  |  |  |  |  |  |  |
|  | 1 and maximum 8. In order to interpolate this into a scale | | | | | |  |  |  |  |  |  |  |  |
|  | from 0-100 the formula ((Total average - 1) * (100/7)) was | | | | | |  |  |  |  |  |  |  |  |
|  | applied.) |  |  |  |  |  |  |  |  |  |  |  |  |  |
|  | **Scale 0-100:** |  |  |  | **Sum:** | |  |  |  | 35,49 |  |  |  |  |
|  |  |  |  |  |  |  |  |  |  |  |  |  |  |  |

**Table text:**

The questionnaire was filled in by the patient in private. Each question, being a statement that the patient would agree to in a higher or lower degree, was provided with a VAS-scale for the patient to fill in. The sums in this example are calculated by adding the score of each question in the actual section, and dividing by the number of questions. The results of question nr 3 was reversed by the formula Result = (11-given answer), to comply with the other sections were increasing number indicates increasing suffering.
